# Mineralogy and Stable Isotopes of Tetradymite from the Dashuigou Tellurium Deposit, Tibet Plateau, Southwest China

**DOI:** 10.1038/s41598-020-61581-3

**Published:** 2020-03-13

**Authors:** Jianzhao Yin, Hongyun Shi

**Affiliations:** 1 Postdoctoral, Chinese Academy of Geological Sciences, Beijing, China; 2 Ph.D., Bureau Veritas Commodities Canada Ltd., Richmond, V7A 4V5 BC Canada

**Keywords:** Economic geology, Mineralogy

## Abstract

Due to the very limited quantity of associated tetradymite, both mineralogical and geochemical research on tetradymite is scarce and incomplete. By taking advantage of the discovery of the Dashuigou tellurium deposit in Tibet Plateau, the authors conducted mineralogical studies on tetradymite. The authors present new mineralogical data including reflectance, micro-pressure hardness, chemical compositions focusing on its chemical formula Bi_2.00_Te_1.89_S_0.95~1.00_, unit cell parameters (*a* = 4.239 Å, *c* = 29.595 Å) and lattice parameters (*a* = 10.172 Å, *v* = 154.391 Å^3^), pyroelectricity (N type), and stable isotopes including sulfur and lead. The authors find that: δ^34^S‰ of tetradymite varies between −0.5~2.1 with a 15-sample average of 0.56, similar to that of meteorites and rocks from the mantle, indicating that the sulfur is from the mantle; lead isotopes of the tetradymite formed in the late metallogenic epoch is different from that of both pyrite and pyrrhotite formed in the early metallogenic epoch, further indicating that the three minerals formed in different metallogenic epochs; lead isotope compositions reveal that tellurium ore bodies emplaced in a quick process mainly in the form of ore magma; lead of the deposit is primarily from the mantle with some captured from the Earth’s crust. These findings help fill in large gaps of information for the mineral tetradymite.

## Introduction

Tellurium (Te) is usually categorized as a scattered or dispersed element (abbreviated as SM). SM are those metals, semimetals and/or nonmetals that have similar geochemical characteristics with Clark values too low to enrich into independent deposits, but that play very important roles in modern science, industry, national defense and the frontiers of technology. It is thought in the traditional theory of mineral deposits and geochemistry that Te could not form independent deposits, but only exist as associated components in other metallic deposits. The abundance of Te in the Earth’s Crust is very low. According to Li^1^, the average content of Te in the Earth’s crust is 2.0 × 10^−8^ in China, and only 1.34 × 10^−9^ worldwide.

At present, the world’s supply of refined tellurium is mainly recovered from Te-bearing minerals including pyrite, sphalerite, chalcopyrite, galena, pyrrhotite, volcanogenetic sulfur, bismuthinite, arsenopyrite, and cassiterite, etc. Generally speaking, only sulfide ores containing more than 0.002% Te can be used. As a result, the amount of refined tellurium that can be recovered is very limited. Most of the recoverable Te in the world is from copper deposits, and it is estimated that only 0.065 kg of Te can be produced in the refining process of one ton of copper^2,3^.

## Regional Geological Setting

**Figure 1:**
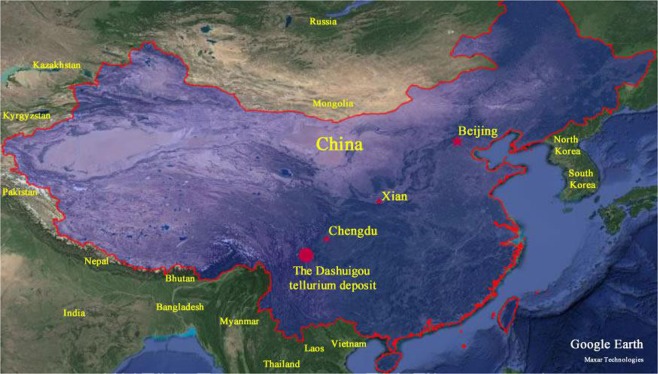
Location map of the dashuigou tellurium deposit. (Google Earth 7.1.8.3036 (32-bit): http://download.pchome.net/industry/geography/detail-20351.html, and 91 v17.5.8: www.91weitu.com; accessed January 25–27, 2020).

The Dashuigou tellurium deposit is located in the transitional belt between the Yangtze Platform and Songpan-Ganzi folded belt, as part of the Tibetan Plateau (Fig. 1). The deposit is nestled in the convergence between the Indian, Eurasian and Pacific Plates. The crust-mantle structures and properties in the region are the result of tectogenesis throughout various geological times. It implies the turning boundary of the Earth’s crust’s thickness. It is also a gravity gradient zone which controls not only the production and development of earthquakes and tectonomagmatic events, but also the distribution of a series of mineral deposits. Geophysical data indicate that the upper mantle below the region uplifts obviously. As a result, the area has characteristics of high heat flow.

There is also a low-velocity, low resistivity zone in the middle crust that is interpreted as a decollement. The abnormal mantle exists under the crust in the region. It has properties of both geosyncline and platform, as well as special characteristics of its own. The belt is a geo-tectonically active zone with very complicated igneous rock structures. According to the regional geophysical data, the region’s characteristics exhibit high velocity, high density, high resistance, high geothermal flow, high magnetism as well as well-developed earthquakes and mantle’s uplift. In summary, this region is both geologically very active and a very important south-north trending tectonomagmatic-mineral belt^3–5^.

The strata, igneous rocks and structures trend south-northward. The strata are low-grade metamorphic rocks of the Silurian, Devonian, Permian systems and middle-lower Triassic series. A large amount of Archaean metamorphic rocks of the Kangding Group emerge to the southeast of the deposit. The well-developed igneous rocks in the region include ultrabasic, basic, neutral, acid and alkaline, produced in different geological times. Different types of mineral resources in the region are very rich; many of these are well known, including Ti, V, Cu, Pb, Zn, SM, REE, coal, asbestos and the Panzhihua Vanadium Titano-magnetite deposit^3–5^.

## Geology of the Deposit

The strata of the area are low-grade metamorphic rocks of the lower-middle Triassic age, including marble, slate and schist. The main wall-rocks of the ore bodies are schist and slate. All of the Triassic strata make up a NNE-trending dome. The geological and geochemical characteristics in the area indicate that the protolith of the tellurium ore veins’ direct wall-rocks is poorly differentiated mantle-derived basalt^3–7^.

Both faults and folds are well-developed in the area. The annular and linear structures together make up special “Ø” pattern structures, which control the formation of different types of endogenetic mineral deposits, including the Dashuigou tellurium deposit.

No intrusive rocks emerge within a 5 km radius around the deposit. Only two small Permian ultrabasic-basic rock bodies emerge within a 10 km range of the deposit. Large neutral, acid and alkaline intrusive bodies exist beyond 10 km, which are unrelated to the deposit.

Quantitative chemical analyses of Te, Bi, Se, As, Au, Ag, Cu, Pb and Zn were conducted on different rock samples including granites, metamorphic rocks, altered rocks, and carbonate veins of different geological times. The main findings are summarized below^3–9^.

The Te content in the granites is under 1 × 10^−7^, which is similar to its Clark value in the Earth’s crust. Te in the metamorphic rocks is slightly higher than in the granites and varies slightly between metamorphic rocks of different geological times, while being relatively higher in the Triassic metamorphic rocks. Of the metamorphic rocks in the same geological time, the Te content in the slate and schist is higher than in the marble. Te content in rocks of the same stratohorizon of the same geological time also varies; namely, it is higher in rocks within the mining area than in those beyond the mining area. Te content is closely related to the intensity of alterations; that is, the ore-forming elements are not derived from the country rocks, but instead from the mantle.

The deposit is located at the northeastern end of the Triassic metamorphic dome. The ore bodies are controlled by and fill a group of shear fractures. Nine tellurium ore veins have been discovered, which strike from 350 to 10 degrees and dip at 55 to 70 degrees westward. Widths of the ore bodies vary between 25 and 30 cm. The narrow ore bodies are in the shape of lenticular veins and have sharp contact with the wall rocks.

The altered rocks occur in narrow bands ranging between several centimeters and one meter in thickness. Altered zones beside the massive ore veins are narrower, at only several centimeters wide. The dominant alterations include dolomitization, silicification, biotitization, muscovitizaion, tourmalinization, sericitizaion, greisenization, and chloritization.

Approximately thirty minerals are identified in the ore, which include tetradymite, pyrrhotite, pyrite, dolomite, quartz, chalcopyrite, tsumoite (BiTe), tellurobismuthite (Bi_2_Te_3_), galena, magnetite, gold, silver, electrum, ilmenite, calcite, calaverite, siderite, mannesite, rutile, muscovite, biotite, sericite, hornblende, chlorite, plagioclase, K-feldspar, tourmaline, hematite, garnet, apatite, and epidote. The first five minerals are the most important and comprise 85% of the ore^3–9^, though tetradymite is so rare that many monographs on mineralogy do not have any related data on it^10–12^.

**Figure 2:**
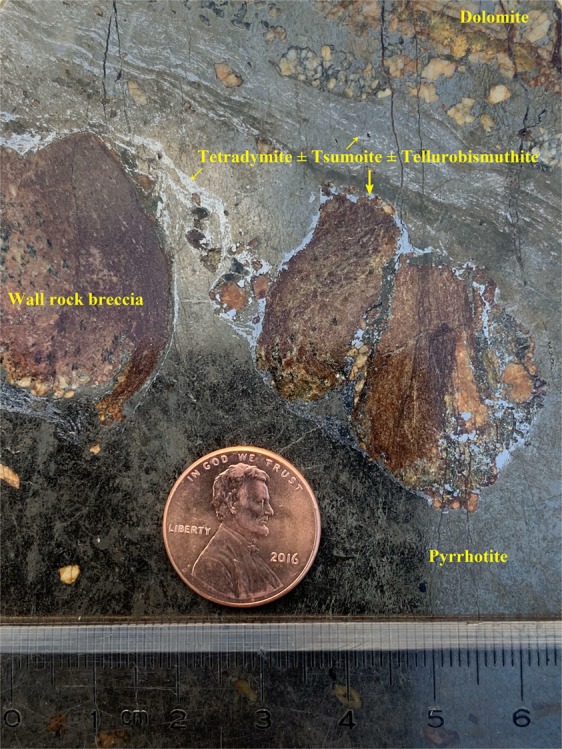
Lead grey-silvery colored tetradymite ± tsumoite (BiTe) ± tellurobismuthite (Bi_2_Te_3_) fine veinlets in massive pyrrhotite (dark colored) + dolomite (brownish white) + wall rock (dark brown) from the deposit (sample #: SD34, Ore body #I-1 in Drift 3).

**Figure 3:**
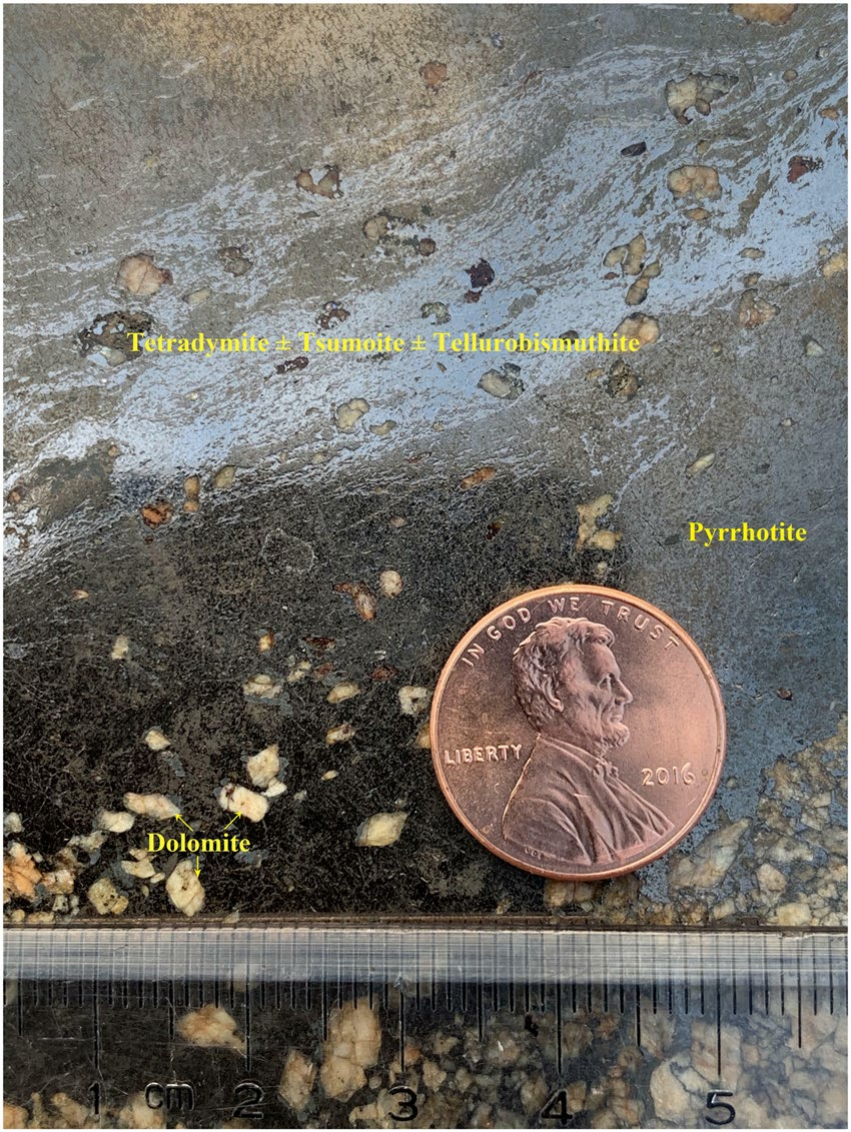
Lead grey-silvery colored tetradymite ± tsumoite (BiTe) ± tellurobismuthite (Bi_2_Te_3_) fine veinlets in massive pyrrhotite (dark colored background) + dolomite (brownish white) from the deposit (sample #: SD40, Ore body #I-1 in Drift 3).

Replacement, remnant, reaction border, and granular are the dominating textures of the ore. Massive, vein/veinlet, stockwork veins are the dominating structures of the deposit (Figs. 2 and 3).

The most important ores are massive and the secondary ores are disseminated. The Te content in the ore varies between 0.01% and 34.58%.

Two mineralization epochs and five stages exist in the deposit:

- Pyrrhotite epoch (177.7~165.1 Ma): including three mineralization stages: carbonate stage (I) → pyrrhotite stage (II) → chalcopyrite stage (III) (from early to late);
- Tellurium epoch (91.71~80.19 Ma): including two mineralization stages, namely: tetradymite stage (I) → tsumoite (BiTe_0.97_) stage (II)^3,4,13,14^.

## Mineralogy of Tetradymite

### Mineragraphy

Tetradymite is the most common telluride and makes up more than 90% of all the tellurides of the deposit. It occurs as a silvery-white fine- to coarse-grained flake with one group of complete cleavages and lower Moh’s hardness (1.4 ~ 2.1). Reflectance of the tetradymite under the four standard wavelengths is listed in Table 1 with a reflectance color of yellowish white. Table 2 lists its micro-pressure hardness.

**Table 1:** Reflectance (R) of tetradymite from the Dashuigou tellurium deposit.

wave length (nm)	470	546	589	650
sample #	*R*%			
1	49.7	51.5	51.3	51.7
2	54.5	55.1	55.8	56.6
3	46.7	47.0	47.5	49.7
Standard sample	black glass: *R*% (air) ~4.5%			

Instrument: German Leitz MPV-3 microscopic photometer.

**Table 2:** Micro-pressure harness (Hv) of tetradymite from the Dashuigou tellurium deposit.

sample #	kg/mm^2^					
	Hv25	Hv50	Hv25	Hv50	average	determination range
1	30.5		37.0		33.8	20.0~80.0
2	38.9	37.1			38.0	the equivalent of
3		66.3		45.3	55.8	1 ~ 2 on Mohs scale

Instrument: German Leitz ORTHO PLAN micro-hardness tester.

In addition to tetradymite, tsumoite (BiTe) and tellurobismuthite (Bi_2_Te_3_) occur as lamellar and myrmekitic intergrowths within tetradymite. These fine-grained minerals are therefore hard to separate (Figs. 2 and 3).

Members of the tetradymite group present complex problems, many of which remain unsolved due to incomplete data^15^.

The group of compounds in the four-compound system Bi-Te-Se-S represents a particular challenge, not least because the number of natural occurrences of the phases that have been comprehensively documented remains limited^16^. The difficulties are compounded by the rarity of the species, the varying quality and limited quantity of published data including reflectance and micro-pressure hardness of tetradymite, and by the invariably smaller-grained size and intergrown character of the mineral. Since the published data on reflectance and micro-pressure hardness of tetradymite is not plentiful, it does not permit a comparison and confirmation regarding the normality of these results.

### Chemical compositions & formula

Four of the nine tellurium veins in total had been mined out by the time this research started. As a result, all samples were collected from the five remaining veins. Chemical compositions of the tetradymite analyzed by electronic probe are listed in Table 3.

**Table 3:** Electron-microprobe data of tetradymite from the Dashuigou tellurium deposit (%).

sample #	Vein	Te	Bi	S	Fe	Cu	As	Ni	Zn	Au	Ag	Total	chemical formula
SD34	# I-4	34.75	60.29	4.57	0.03	0.04	0.09	0.01	0.09	0.01	0.02	99.90	Bi_2.00_Te_1.89_S_0.99_
SD36	# I-5	34.80	60.24	4.42	0.03	0.02	0.10	0.02	0.06	0.02	0.04	99.75	Bi_2.00_Te_1.89_S_0.96_
SD41	# I-1	34.54	60.22	4.81	0.13	0.04	0.00	0.02	0.06	0.03	0.04	99.89	Bi_2.00_Te_1.88_S_1.04_
SD45	# I-2	34.38	59.48	4.78	0.03	0.04	0.00	0.15	0.10	0.01	0.08	99.05	Bi_2.00_Te_1.89_S_1.05_
SD53	# I-10	34.58	60.03	4.71	0.14	0.05	0.05	0.09	0.10	0.03	0.02	99.80	Bi_2.00_Te_1.89_S_1.02_
SD59	# I-10	34.57	60.21	4.38	0.09	0.05	0.05	0.09	0.11	0.03	0.00	99.58	Bi_2.00_Te_1.88_S_0.95_
**average**		34.60	60.08	4.61	0.08	0.04	0.05	0.06	0.09	0.02	0.03	99.66	Bi_2.00_Te_1.89_S_1.00_

Based on the results presented in Table 3, chemical compositions of the tetradymite samples collected from different ore bodies of the mine are very similar: Te content varies between 34.38~34.80% with a maximum difference of 0.42% and average of 34.60%; Bi content varies between 59.48~60.29% with a maximum difference of 0.81% and average of 60.08%; S content varies between 4.38~4.81% with a maximum difference of 0.43% and average of 4.61%.

Synthetic tetradymite (Bi_2_Te_1.9_S_1.1_), which is similar to the tetradymite from the Dashuigou deposit, and two compounds, Bi_48_Te_21_S_31_ and Bi_5_Te_3_S_2_, were generated at 400 °C. Sulfur-rich tetradymite appears more chemically stable than stoichiometric Bi_2_Te_2_S^16^.

The contents of trace elements in the tetradymite are similar to those in the mine’s pyrrhotite and pyrite. The only difference is tetradymite is richer in gold than both the pyrite and pyrrhotite, which is identical to the observation results under microscope study of the minerals. This indicates that gold formed in the tellurium epoch mentioned above.

### X-Ray power diffraction data

Due to insufficient quantity of the tetradymite mineral, single-crystal X-ray studies are lacking and previous X-ray diffraction data and unit cell parameters of tetradymite were mainly obtained via crystal powder photography, the accuracy of which is not very satisfactory.

The quantitative phase analysis of one powder sample (#SD-40, Fig. 3) using the RIETVELD method and X-ray powder diffraction data, Project 1902410 – PO# 18576, was done by Jacob Kabel, and Dr. Elisabetta Pani, *et al*. from Dept. of Earth, Ocean & Atmospheric Sciences, the University of British Columbia, Canada in December, 2019.

#### Experimental method and procedure

The sample SD-40 was reduced to the optimum grain-size range for quantitative X-ray analysis (<10 μm) by grinding under ethanol in a vibratory McCrone Micronizing Mill for 10 minutes. Continuous-scan X-ray powder-diffraction data were collected over a range 3–80 °2 with CoKα radiation on a Bruker D8 Advance Bragg-Brentano diffractometer equipped with an Fe filter foil, 0.6 mm (0.3°) divergence slit, incident- and diffracted-beam Soller slits and a LynxEye-XE detector. The long fine-focus Co X-ray tube was operated at 35 kV and 40 mA, using a take-off angle of 6°.

#### Results

**Figure 4:**
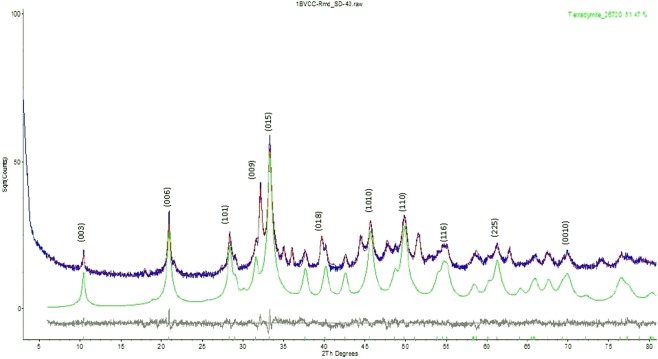
Rietveld refinement plot of sample SD-40 (blue line - observed intensity at each step; red line - calculated pattern; solid grey line below - difference between observed and calculated intensities; vertical bars - positions of all Bragg reflections).

The X-ray diffractogram was analyzed using the International Centre for Diffraction Database PDF-4 and Search-Match software by Bruker. X-ray powder-diffraction data of the sample were refined with Rietveld program Topas 4.2 (Bruker AXS). The results of quantitative phase analysis by Rietveld refinements are given in Table 4. These amounts represent the relative amounts of crystalline phases normalized to 100%. The Rietveld refinement plot is shown in Fig. 4. Lattice parameters and volumes are given in Table 5.

**Table 4:** Results of quantitative phase analysis (wt.%) – project 1902410 – PO# 18576.

Mineral	ICSD Collection Code	Ideal Formula	SD-40
Tetradymite	26720	Bi_2_Te_2_S	51.5
Tsumoite	100654	BiTe	11.5
Tellurobismuthite	74348	Bi_2_Te_3_	1.9
Pyrrhotite 4 C	42491	Fe_7_S_8_	26.1
Ankerite – Dolomite	66333	Ca(Fe^2+^, Mg, Mn)(CO_3_)_2_− CaMg(CO_3_)_2_	8.1
Chalcopyrite	80095	CuFeS_2_	0.5
Quartz	174	SiO_2_	0.4
Total			100.0

**Table 5:** Results of quantitative phase analysis (wt.%) XRD-Rietveld and lattice parameters.

Phase Name	ICSD Collection Code	SG	Refined Values						
			wt.%	a (Å)	b (Å)	c (Å)	alpha(°)	beta(°)	V (Å^3^)
Tetradymite	26720	R-3R	51.5	10.172			24.113		154.391
Tsumoite	100654	P-3m1	11.5	4.432		23.995			408.294
Tellurobismuthite	74348	R-3mH	1.9	4.397		30.512			510.975
Pyrrhotite	42491	C12/c1	26.1	11.935	6.864	12.834		117.236	934.840
Dolomite	66333	R-3H	8.1	4.679		16.573			314.297
Chalcopyrite	80095	I-42d	0.5	5.295		10.402			291.630
Quartz low	174	P3221S	0.4	4.929		5.361			112.811
Total			100.0						

**Figure 5:**
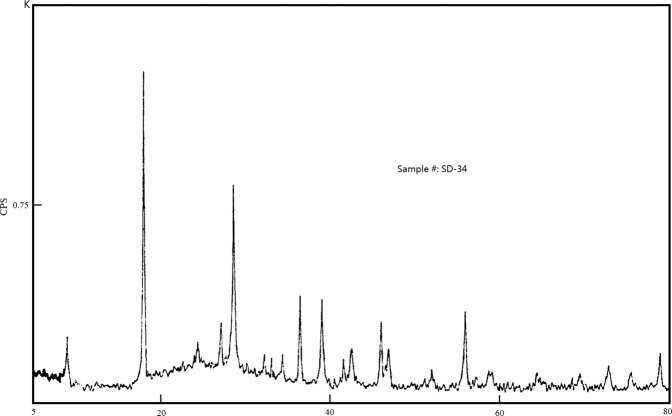
X-ray crystal power diffractogram of tetradymite from Dashuigou deposit.

Another X-ray crystal powder diffraction analysis done in lab of the Research Center of Standard Materials, China Academy of Metrological Sciences, Beijing of China reveals that the tetradymite from the Dashuigou tellurium deposit is different than that from the Paonia mine in Colorado, US, but similar to kawazulite from the Kawazu mine in Japan. Even so, tetradymite from the Dashuigou deposit is lacking in Se compared to the Japanese kawazulite (Tables 6 and 7 and Fig. 5).

**Table 6:** Comparison of X-ray diffraction data of tetradymite from Dashuigou deposit and tetradymite/kawazulite from deposits of other countries.

Item	tetradymite		tetradymite		kawazulite	
	Bi_2.00_Te_1.89_S_1.00_		N/A		Bi_2.07_Te_1.95_Se_0.97_S_0.05_	
	the Dashuigou mine		JCPDS 19–1330		JCPDS 29–248	
	Sichuan Province, China		Paonia, Colorada, USA		the Kawazu mine	
sample source	Nat. Museum of China		U.S.Nat. Museum R-395		Shizuoka, Japan	
	*d* (Å)	I/I_0_	*d* (Å)	I/I_0_	*d* (Å)	I/I_0_
	9.973	15			9.900	10
	4.940	90	4.860	30	4.920	40
	3.651	16	3.610	30	3.640	30
	3.295	19	3.260	30	3.300	10
	3.123	100	3.100	100	3.120	100
	2.776	12	2.756	30	2.780	10
	2.609	12	2.592	30	2.610	20
	2.464	31	2.453	30	2.460	10
	2.305	43	2.292	100	2.310	50
	2.169	14	2.159	50	2.170	10
	2.123	27	2.111	75	2.120	50
	1.971	27	1.965	75	1.977	10
	1.938	21	1.929	75	1.933	10
	1.832	5	1.825	30	1.832	10
	1.783	6	1.776	30	1.757	20
	1.755	14	1.743	50		
	1.683	4	1.679	10		
	1.644	32	1.638	756	1.648	10
	1.607	8	1.603	50	1.609	6
	1.569	13	1.569	50	1.571	10
			1.556	50	1.560	10
	1.517	4	1.513	10		
	1.445	10	1.441	50	1.447	10
X-ray	1.428	6	1.426	30		
diffraction	1.408	4	1.407	10		
data	1.386	4	1.383	10		
	1.370	5	1.369	30		
	1.353	11	1.348	75	1.352	10
	1.300	15	1.297	75	1.303	10
	1.262	10	1.258	506		
	1.234	5	1.232	10		
			1.222	10		
	1.213	18	1.212	75		
	1.187	5	1.184	30		
	1.172	3	1.172	10		
	1.151	3				
	1.113	2	1.108	10		
	1.097	4	1.096	10		
	1.086	4	1.085	30		
	1.054	6	1.053	50		
	1.041	4				
	1.015	13	1.015	75		
			1.003	30		
	0.982	5	0.986	30		
			0.973	10		
	0.965	5	0.962	30		
	0.954	4				
	0.945	6	0.943	50		
	0.931	4				
	0.915	7	0.915	50		
	0.907	4	0.906	10		
	0.896	8	0.896	50		
			plus 3 lines to 0.8258			
	CuKa		CuKa		CuKa	
	voltage: 40 kV		l = 1.5418 Å		l = 1.5418 Å	
test conditions	electricity: 50 mA		Ni filtration		Ni filtration	
	slit: DS = SS = 1, RS = 0.15		camera diameter: 14.3 mm		camera diameter: 57.3 mm	
	scanning speed: 2 deg/min					
	step: 0.02 deg					
crystal system	hexagonal system		hexagonal system		hexagonal system	
unit cell	*a* = 4.239		*a* = 4.238		*a* = 4.240	
parameters	c = 29.595		c = 29.589		c = 29.660	
space group	*R*3*m, 3 2/m*		*R*3*m, 3 2/m*		*R*3*m, 3 2/m*

**Table 7:** Unit cell parameters of tetradymite from Dashuigou deposit and its comparisons with tetradymite/kawazulite from other countries.

series #	sample #	location	unit cell parameters			note
		vein #	*a* (Å)	*c* (Å)	*v* (Å^3^)	
1	SD34	# I-4	4.236	29.594	459.943	
2	SD36	# I-5	4.239	29.651	461.424	
3	SD40	# I-1	4.261	29.641	461.710	
4	SD46	# I-2	4.241	29.538	460.038	instrument:
5	SD58	# I-10	4.239	29.595	460.594	Rigaku D/max-rC
6	SD59	# I-10	4.248	29.582	462.366	
7	SD61	# I-10	4.238	29.569	459.830	
average			4.240	29.596	460.843	
tetradymite	USNM	Paonia	4.238	29.589	N/A	according to
	R-395	Cololado, USA				JCPDS 19–1330
kawazulite		Kawazu mine, Japan	4.240	29.660	N/A	JCPDS 29–248

test conditions of tetradymite from the Dashuigou mine:

CuK*a* radiation, voltage: 40 kV, electricity: 50 mA,

slit: DS = SS = 1, RS = 0.15, scanning speed: 2 deg/min, step: 0.02 deg, smooth count: 11.

Kawazulite, Bi_2_Te_2_Se, which was originally described by Kato (1970) as the Se analogue of tetradymite, is isostructural with tetradymite, which has been synthesized by Glaz (1967), Evdokimenko and Tsypin (1971), and Abrikosov and Beglaryan (1973). The compositional limits of tetradymite have been known to be Bi_2_Te_2_S-Bi_2_Te_1.7_S_1.3_As determined by Kuznetsov and Kanishcheva (1970). Pauing (1975) explained why the substitution of Te by S increases the chemical stability. The calculated intensities of an X-ray powder-diffraction pattern of kawazulite with the ordered tetradymite structure type are similar to the visually estimated observed X-ray powder-diffraction intensities of kawazulite (PDF 29–248). The visually estimated observed intensity data is not sufficiently accurate to exclude partial ordering of Se and Te^15^. Extensive solid-solution between S- and Se-bearing end members can be documented in many series; for instance, tetradymite – kawazulite, and continuous solid-solution between tetradymite and kawazulite is well developed^16^.

The experimental and calculated two theta values with hkl indices are listed in Table 8.

**Table 8:** The hkl vs two theta of the tetradymite from the Dashuigou tellurium deposit*.

Tetradymite (ICDD 00-42-1447)						λ	Co tube
							1.79
No.	h	k	l	d [Å]	I [%]		2θ [°]
1	0	0	3	9.87	2		0.00
2	0	0	6	4.929	10		0.00
3	1	0	1	3.644	20		0.00
4	0	1	2	3.566	2		0.00
5	0	0	9	3.288	10		0.00
6	0	1	5	3.121	100		0.00
7	1	0	7	2.77	5		0.00
8	0	1	8	2.606	5		0.00
9	0	0	12	2.4655	1		0.00
10	1	0	10	2.3033	20		0.00
11	0	1	11	2.17	5		0.00
12	1	1	0	2.1199	40		0.00
13	1	1	3	2.0723	1		0.00
14	0	0	15	1.9709	2		0.00
15	1	1	6	1.9473	10		0.00
16	1	0	13	1.9335	10		0.00
17	0	1	14	1.832	3		0.00
18	2	0	2	1.8232	1		0.00
19	1	1	9	1.7813	3		0.00
20	2	0	5	1.7538	20		0.00
21	0	2	7	1.6839	2		0.00
22	1	0	16	1.6512	1		0.00
23	0	0	18	1.6434	3		0.00
24	1	1	12	1.6068	5		0.00
25	0	1	17	1.5721	1		0.00
26	0	2	10	1.5598	10		0.00
27	2	0	11	1.5161	2		0.00
28	1	1	15	1.4435	5		0.00
29	0	2	13	1.4286	3		0.00
30	2	0	14	1.3861	2		0.00
31	1	2	2	1.3823	1		0.00
32	0	1	20	1.3716	1		0.00
33	2	1	4	1.3634	1		0.00
34	1	2	5	1.3508	20		0.00
35	2	1	7	1.3184	1		0.00
36	1	1	18	1.2982	10		0.00
37	1	0	22	1.2626	1		0.00
38	2	1	10	1.2561	10		0.00
39	0	0	24	1.2329	2		0.00
40	3	0	0	1.2237	10		0.00
41	0	1	23	1.2133	5		0.00
42	3	0	6	1.1876	2		0.00
43	2	1	13	1.1844	5		0.00
44	1	1	21	1.1719	1		0.00
45	1	2	14	1.1587	1		0.00
46	2	0	20	1.1513	1		0.00
47	3	0	9	1.1475	1		0.00
48	2	1	16	1.1095	1		0.00
49	0	0	27	1.0954	1		0.00
50	1	2	17	1.0846	1		0.00

*Barry Whittington, Senior Mineralogist - Pacific Zone, Bureau Veritas Minerals Pty Ltd, helped with the hkl calculation.

### Pyroelectricity

It is believed that pyroelectricity of minerals can be used to determine their origin and process of formation. Unfortunately, there existed no pyroelectricity data of tetradymite prior to the research of this paper.

Pyroelectricity of minerals can be divided into N (electron) type, P (electron hole) type and the mixed N and P type.

Pyroelectricity characteristics of the tetradymite from Dashuigou deposit are listed in Table 9.

**Table 9:** Pyroelectricity of tetradymite from the Dashuigou tellurium deposit.

sample #	location	TVG*	SVp	SVn	SVnp	pyroelectricity	conduction	EHS/TS**
	vein #	mV	mV	mV	mV	a(µV/°C)	type	
SD34	# I-4	(−34.1~−23.0)	0	−292.6	−29.26	−209.0	N	0/10
SD36	# I-5	(−42.0~−30.7)	0	−364.0	−36.40	−260.0	N	0/10
SD40	# I-1	(−40.9~−33.2)	0	−383.6	−38.36	−274.0	N	0/10
SD46	# I-2	(−38.8~−34.2)	0	−368.4	−36.84	−263.14	N	0/10
SD58	# I-10	(−37.0~−29.5)	0	−334.6	−33.46	−239.0	N	0/10
SD59	# I-10	(−32.8~−27.1)	0	−293.6	−29.36	−209.71	N	0/10
SD61	# I-10	(−41.2~−33.5)	0	−345.8	−34.58	−247.0	N	0/10
**average**		(−38.11~−29.89)	0	−304.4	−30.04	−243.12	N	0/10
**excitation temperature**	140 °C							

Note: TVG* = thermoelectric voltage range, EHS/TS** = electron hole sample/total sample.

The tetradymite from Dashuigou deposit is completely of N conduction type. All of its pyroelectricity is negative, and the values are both close to each other and vary between −209.0 ~ −274.0 µV/°C. With a maximum difference of −65.0 µV/°C and an average of −243.12 µV/°C, the data implies that the tellurium veins were all formed in the same geological event and from the same source.

The negative pyroelectricity of the tetradymite resulted from insufficient sulfur, As and Se impurities, and other isomorphous mixtures of Te in tetradymite.

## Sulfur and Lead Isotopes of Tetradymite

### Sulfur isotope

**Figure 6:**
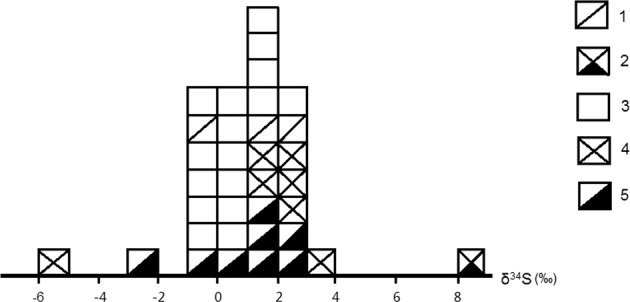
Histogram of δ^34^S_CDT_ (‰) of the dominant sulfides from the Dashuigou deposit. (35 samples in total included in Table 10). 1. chalcopyrite, 2. chalcocite, 3. tetradymite, 4. pyrite, 5. Pyrrhotite.

Sulfur isotope results of the dominant sulfides collected from various veins of the deposit are provided in Table 10. It can be seen that sulfur isotopes of various sulfides formed in different veins of different metallogenic epochs and/or stages are very close to each other, varying within a narrow range with an average below 1‰, a crest value around 0.6‰, and a clear tower effect (Fig. 6).

**Table 10:** Sulfur isotope results of the sulfides from the Dashuigou tellurium deposit.

series #	sample id	sample name	location	δ^34^S_CDT_ (‰)	note
1	SD-10	pyrrhotite	from a Pyr vein between #II & III Pyr Veins	−3.1	
2	SD-15		from #III-1 Pyr Vein	−1.4	
3	SD-23		from #I-4 Tellurium Vein	1.2	
4	SD-29		from #I-5 Tellurium Vein	2.1	
5	SD-41		from #I-1 Tellurium Vein	−0.1	
6	SD-55		from #I-10 Tellurium Vein	1.6	
7	SD-65		from #I-8 Tellurium Vein	0.6	
8	SD-71		from a Pyr vein of #IV Ore Zone	0.5	
9	SD-17	pyrite	from #III-2 Pyr Vein	1.4	scope*
10	SD-23		from #I-4 Tellurium Vein	1.7	(−3.1‰ ~
11	SD-29		from #I-5 Tellurium Vein	2.0	2.8‰)
12	SD-41		from #I-1 Tellurium Vein	1.6	
13	SD-52		from a DV at marble-schist contact of the deposit	2.8	
14	SD-55		from #I-10 Tellurium Vein	1.4	
15	SL-06		from a QV next to the deposit	−0.6	range*
16	SL-10		from the metamorphosed basalt next to the deposit	−5.7	5.9‰
17	SD-34	tetradymite	from #I-4 Tellurium Vein	−0.2	
18	SD-40		from #I-1 Tellurium Vein	0.6	
19	SD-46		from #I-2 Tellurium Vein	0.5	
20	SD-59		from #I-10 Tellurium Vein	1.4	average*:
21	SD-36		from #I-5 Tellurium Vein	0.3	0.72‰
22	SL-23	chalcocite	from a copper showing next to the deposit	8.2	
23	SD-10	chalcopyrite	from a Pyr vein between #II & III Pyr Veins	−1.4	
24	SD-15		from #III-1 Pyr Vein	1.9	
25	SD-71		from a Pyr vein of #IV Ore Zone	0.5	
26	TB1	tetradymite	free pickup from the ore stockpile	0.4	
27	TB2		free pickup from the ore stockpile	0.4	
28	TB3		free pickup from the ore stockpile	2.1	
29	TB4		free pickup from the ore stockpile	0.2	
30	TB5		free pickup from the ore stockpile	−0.5	
31	TB6		free pickup from the ore stockpile	0.3	
32	TB7		free pickup from the ore stockpile	0.8	
33	TB8		free pickup from the ore stockpile	0.5	
34	TB9		free pickup from the ore stockpile	0.5	
35	TB10		free pickup from the ore stockpile	1.1	

Note: Pyr-pyrrhotite, DV-dolomite vein, QV-quartz vein, *only counting those samples collected within the deposit.

Cao and Luo researched and published their δ^34^S_CDT_‰ results of 1.13~3.17 with an average of 1.96^17^, which indicated that sulfur isotopes of the Dashuigou deposit were very homogeneous and thus originated from the deep mantle.

In general, sulfur isotopes of the deposit are very similar to those of meteorite, moon rock and mantle-derived materials, indicating that the sulfurs are from the mantle. This finding is in agreement with Cao and Luo’s studies^17^. The δ^34^S_CDT_‰ of series #16 & #22 samples in Table 10, which are collected from regional country rocks far from the deposit, clearly deviate from that of samples collected from the deposit.

The series #16 sample is coarse- to very coarse-grained cubic pyrite in the upper Permian metamorphosed basalt to the south of the deposit, while the series #22 sample is chalcocite from the Shaoyaocao copper showing, demonstrating a connection to the basic to ultra-basic intrusive to the east of the deposit. In theory, δ^34^S_CDT_‰ of these two samples should be close to that of mantle-derived materials (−3 ~ 2), since their wall rocks are from the mantle. In fact, they are not similar to each other, which may be owed to the post metamorphism after their formation.

δ^34^S_CDT_‰ of all the pyrrhotite samples varies between −3.1 ~ 2.1 with an 8-sample average of 0.175, close to that of the meteorite, indicating that they are mantle derived. Meanwhile, δ^34^S_CDT_‰ of all the pyrite samples is between 1.4 ~ 2.8 with a 6-sample average of 1.717, also close to that of meteorite and implying that they too are mantle derived.

δ^34^S_CDT_‰ of both the deposit’s pyrrhotite and pyrite formed in the same metallogenic epoch are in a similar narrow scope. For samples #SD-29 and SD-55 in Table 10, δ^34^Spyrite‰ <δ^34^Spyrrhotite‰, suggesting that the sulfur exchange between pyrite and pyrrhotite of these samples did not reach balance. For samples #SD-23 and SD-41 in Table 10, δ^34^Spyrite‰> δ^34^Spyrrhotite‰, indicating that sulfur exchange between pyrite and pyrrhotite of these samples reached balance.

As shown in Table 10 and briefly discussed above, δ^34^S_CDT_‰ of both pyrrhotite and pyrite varies within a very narrow range, indicating that sulfur exchange between pyrite and pyrrhotite became highly uniform and achieved balance. As a result, δ^34^S_Σs_‰, the general sulfur component of the deposit’s metallogenic hydrothermal solution, can be calculated by the following equation^18^:

$${\delta }^{34}{{\rm{S}}}_{\Sigma {\rm{s}}}={\Sigma \delta }^{34}{{\rm{S}}}_{i}={{\rm{X}}}_{1}{\delta }^{34}{{\rm{S}}}_{1}+{{\rm{X}}}_{2}{\delta }^{34}{{\rm{S}}}_{2}+\bullet \bullet \bullet \bullet \bullet \bullet $$

For the pyrrhotite (Pyr) and pyrite (Py) veins of the deposit’s early metallogenic epoch, the specific equation should be as follows:

$${{\rm{\delta }}}^{34}{{\rm{S}}}_{\Sigma {\rm{s}}}={{\rm{X}}}_{1}{{\rm{\delta }}}^{34}{{\rm{S}}}_{{\rm{Pyr}}}+{{\rm{X}}}_{2}{{\rm{\delta }}}^{34}{{\rm{S}}}_{{\rm{Py}}}$$

Taking X_1_ = 98% (Pyr% by volume of total sulfides in the veins) and X_2_ = 2% (Py% by volume of total sulfides in the veins), average values of δ^34^S_Pyr_‰ and δ^34^S_Py_‰ are respectively 0.175 and 1.817; therefore, δ^34^S_Σs_‰, the general sulfur component of the early metallogenic epoch’s metallogenic hydrothermal solution should be:

$${{\rm{\delta }}}^{34}{{\rm{S}}}_{\Sigma {\rm{s}}}=0.98\times 0.175\textperthousand +0.02\times 1.817\textperthousand =0.208\textperthousand $$

The general sulfur component of the early metallogenic epoch’s hydrothermal solution is close to that of the meteorite and also indicates that the deposit’s sulfur is derived from the mantle.

Sulfur isotopes of tetradymite (Tt) formed in the late tellurium epoch of the deposit vary narrowly between −0.5~2.1‰, with a range value of 2.6‰, a peak value around 0.5‰, and a 15-sample average of 0.56‰, also close to that of the meteorite and mantle materials. Sulfur isotopes of chalcopyrite (Cp) of the late tellurium epoch vary between −1.4‰ and 1.9‰ with a range of 2.3‰ and a 3-sample average of 0.33‰, likewise close to that of the meteorite and mantle materials.

Similarly as above, the general sulfur component δ^34^S_Σs_‰ of the late tellurium epoch’s metallogenic hydrothermal solution can be calculated as follows:

$${{\rm{\delta }}}^{34}{{\rm{S}}}_{\Sigma {\rm{s}}}={{\rm{X}}}_{1}{{\rm{\delta }}}^{34}{\rm{STt}}+{{\rm{X}}}_{2}{{\rm{\delta }}}^{34}{{\rm{S}}}_{{\rm{Cp}}}=0.99\times 0.56\textperthousand +0.01\times 0.33\textperthousand =0.56\textperthousand $$

The general sulfur component of the late tellurium metallogenic epoch’s hydrothermal solution is close to that of the meteorite and demonstrates that the deposit’s sulfur is derived from the mantle.

Per discussions on sulfur isotopes of the deposit’s minerals above, the authors’ preliminary conclusions are that δ^34^S of both single sulfide minerals from different veins of different metallogenic epochs and the general total sulfur isotope components of the deposit’s metallogenic hydrothermal solutions vary within very narrow scopes with very small ranges close to 0.0‰. As a result, the deposit’s sulfur is very close to that of the meteorite and may be derived from the mantle.

### Lead isotope

Lead isotope results of samples collected from the study area are listed in Table 11, and further summarized in Table 12. Model lead ages in Table 11 vary significantly between 0.00 ~ 916.25 Ma, likely indicating that the lead isotopes mainly consist of more radioactive lead which is not homogeneous and thus results in strong anomalous model ages.

**Table 11:** Lead isotope results of samples from the study area.

series #	sample id	name	location	^204^Pb	^206^Pb	^207^Pb	^208^Pb	^206^Pb	^207^Pb	^208^Pb	Pb Isotope	M.L.A
				%				^204^Pb	^204^Pb	^204^Pb	a. w.	Ma
1	SD-10	Pyr	between #II & III PV	1.357	24.924	21.418	52.301	18.362	15.779	38.531	207.233	310.00
2	SD-15		from #III-1 PV	1.388	25.791	21.184	51.636	18.580	15.261	37.198	207.217	0.00
3	SD-23		next to #I-4 Ore Vein	1.401	24.980	21.446	52.173	17.833	15.309	37.245	207.230	235.00
4	SD-26		next to #I-4 Ore Vein	1.368	25.251	21.296	52.082	18.464	15.573	38.086	207.227	97.50
5	SD-29		next to #I-5 Ore Vein	1.370	25.066	21.304	52.260	18.291	15.546	38.136	207.231	185.00
6	SD-34		next to #I-4 Ore Vein	1.384	25.262	21.185	52.170	18.251	15.305	37.691	207.228	0.00
7	SD-41		next to #I-1 Ore Vein	1.355	26.382	21.014	51.249	19.473	15.511	37.828	207.208	0.00
8	SD-55		next to #I-10 Ore Vein	1.359	25.624	21.092	51.925	18.860	15.525	38.219	207.222	0.00
9	SD-65		next to #I-8 Ore Vein	1.353	24.753	21.972	51.922	18.294	16.238	38.373	207.231	916.25
10	SD-71		from a PV of #IV OZ	1.383	24.795	21.420	52.403	17.929	15.488	37.892	207.235	360.00
11	SD-17	Py	from #III-2 PV	1.375	24.872	21.399	52.355	18.095	15.568	38.088	207.234	341.25
12	SD-23		next to #I-4 Ore Vein	1.411	24.921	21.463	52.206	17.665	15.214	37.006	207.231	235.00
13	SD-29		next to #I-5 Ore Vein	1.385	25.170	21.297	52.148	18.176	15.379	37.657	207.228	41.25
14	SD-41		next to #I-1 Ore Vein	1.375	24.871	21.407	52.348	18.090	15.570	38.075	207.234	360.00
15	SD-52		from a dolomite vein at marble and ore-bearing schist contact	1.378	25.271	21.260	52.091	18.338	15.428	37.799	207.227	0.00
16	SD-55		next to #I-10 Ore Vein	1.395	24.764	21.528	52.314	17.758	15.437	37.513	207.234	441.25
17	SL-10		from meta-basalt (P_2_^1^) at the Liushapo next to the deposit	1.360	25.002	21.184	52.455	18.386	15.578	38.575	207.234	160.00
18	SD-34	Td	from #I-4 Ore Vein	1.313	24.979	21.190	52.518	19.027	16.142	40.006	207.236	360.00
19	SD-36		from #I-5 Ore Vein	1.383	25.348	21.132	52.138	18.330	15.281	37.703	207.226	0.00
20	SD-40		from #I-1 Ore Vein	1.350	25.205	21.147	52.299	18.675	15.668	38.749	207.230	35.00
21	SD-46		from #I-2 Ore Vein	2.008	24.830	21.877	51.285	12.366	10.895	25.541	207.204	0.00
22	SD-59		from #I-10 Ore Vein	1.338	25.222	21.079	52.361	18.853	15.756	39.139	207.231	47.50
23	SL-01	G(*γ*_5_^1^)	from Niubeishan near the mine	1.319	26.305	20.712	51.664	19.947	15.706	39.177	207.214	0.00
24	SD-60	S (T_1–2_)	from the footwall of #I-10 ore vein	1.371	25.248	21.103	52.279	18.419	15.395	38.139	207.229	0.00

Note: a.w. - atomic weight; M.L.A - model lead age, the following primal lead isotope ratios are used to calculate the model lead.

ages: a_0_ = 9.307, b_0 _= 10.294, c_0_ = 29.476, t_0 _= 4,430 Ma, decay constants λ_1 _= 1.55 × 10^−10^/a, λ_2 _= 9.85 × 10^−10^/a, and λ_3_ = 4.59 × 10^−11^/a.

Pyr - pyrrhotite, Py - pyrite, Td - tetradymite, G - granite, S - slate, PV - pyrrhotite vein, OZ - ore zone.

Lab: Institute of Geology & Geophysics, Chinese Academy of Sciences.

**Table 12:** Summarized characteristics of lead isotopes of samples from the study area.

series #		1	2	3	4	5	6*	7	8
sample		Pyr	Py	Td	G	Py	S + S	M	Bt
note		collected from the deposit			Ƴ_5_^1^	P_2_^1^	T_1–2_	T_1–2_	P_2_
# of sample		10	6	5	1	1	3	1	2
	scope	17.833~	17.665~	12.366~	n/a	n/a	18.419~	n/a	18.175~
		19.473	18.338	19.028			19.616		18.235
^206^Pb/^204^Pb	range	1.640	0.673	6.661	n/a	n/a	1.197	n/a	0.030
	average	18.434	18.020	17.450	19.947	18.386	18.833	19.803	18.205
	scope	15.261~	15.214~	10.895~	n/a	n/a	15.367~	n/a	15.549~
		16.238	15.570	16.142			15.786		15.629
^207^Pb/^204^Pb	range	0.978	0.356	5.247	n/a	n/a	0.419	n/a	0.040
	average	15.554	15.433	14.748	15.706	15.578	15.516	15.818	15.589
	scope	37.198~	37.006~	25.541~	n/a	n/a	38.139~	n/a	38.380~
		38.531	38.088	40.006			43.506		38.490
^208^Pb/^204^Pb	range	1.333	1.082	14.465	n/a	n/a	5.367	n/a	0.053
	average	37.920	37.690	36.228	39.177	38.575	40.202	38.932	38.425

Note: Pyr - pyrrhotite, Py - pyrite, Td - tetradymite, G - granite, S + S - slate & schist, M - marble, Bt - basalt,

* - results of 2 of the 3 sample of series #6 sample are after Wang^19^.

**Figure 7:**
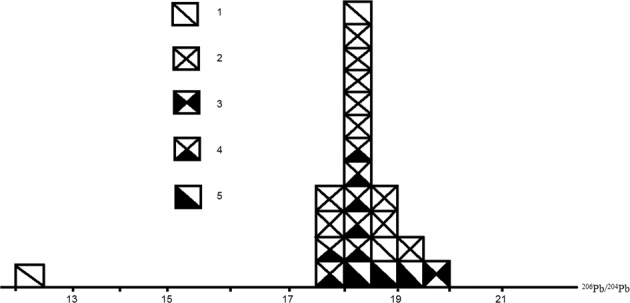
Histogram of ^206^Pb/^204^Pb of samples from the study area. 1. slate, 2. pyrrhotite from the deposit, 3. granite, 4. pyrite from the deposit, 5. tetradymite from the deposit.

Figure 7 shows the components of lead in the study area. According to Zhang^18^, the area to the left of ^206^Pb/^204^Pb = 18.5 is generally the evolutionary area of normal lead, while to the right is the evolutionary area of anomalous leads. This confirms that lead isotopes in the study area, especially those of ore samples from the deposit, are not uniform. As a result, the model lead ages in Table 11 does not make sense geologically.

**Figure 8:**
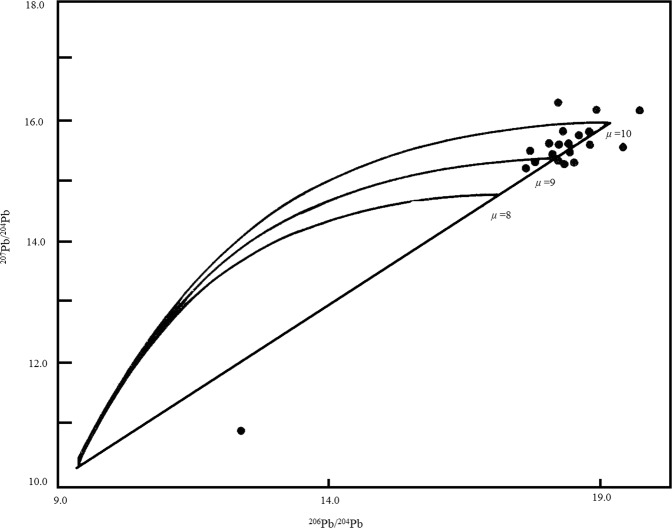
^207^Pb/^204^Pb - ^206^Pb/^204^Pb diagram of samples from the study area. (The single-stage evolution isochron and growth curve diagram of lead isotopes, part of the samples coincide with each other on the diagram).

In general, the lead isotopes of pyrite are more homogeneous than that of pyrrhotite, with the least uniformity in tetradymite. This can be seen in Fig. 8, as some samples fall out of the semilunar area composed of the µ = 10 (0 time) line and the evolutionary curve, indicating that there exists anomalous lead. This further confirms that stable lead isotopes in the area are a mixture of both normal and anomalous ones.

Parent rock of the slate is mantle derived basaltic rock^3,5,6^. A comparison of slate’s lead isotope (series #24 sample in Table 11) with that of material with proven origins is presented in Table 13. It can be seen that the deposit’s wall rock slate is mantle derived, though post metamorphism made it deviate from the lead of pure mantle materials.

**Table 13:** Characteristics of average lead isotopes of both the mantle & the crust.

lead isotopes	^206^Pb	^207^Pb	^208^Pb
	^204^Pb	^204^Pb	^204^Pb
the mantle	18.10	15.42	37.70
the lower crust	17.27	15.29	38.57
the upper crust	19.33	15.73	39.08

Upon synthesizing lead isotope results of slate and schist from the middle-lower Triassic ages published by former researchers in the study area (Table 14), lead isotope deviations caused by the post metamorphism become clear.

**Table 14:** Geological time interpretation of lead isotopes of various rocks.

lead isotope	Precambrian*	Post-cambrian*	research area	
			schist/slate at the mine	granite (Ƴ_5_^1^) outside the mine
^204^Pb	>1.42%	<1.40%	1.371% (1)**	1.319% (1)
^206^Pb	<24.00%	>24.75%	25.248% (1)	26.305% (1)
^207^Pb	>22.00%	<21.50%	21.103% (1)	20.712% (1)
^206^Pb/^204^Pb	>17.00%	>17.75%	18.833% (3)	19.947% (1)
^208^Pb/^204^Pb	>37.00%	>37.50%	40.202% (3)	39.177% (1)
^206^Pb/^207^Pb	<1.19	>1.14	1.200 (1)	1.270 (1)
^208^Pb/^206^Pb	<2.37	<2.11	2.070 (1)	1.960 (1)
^208^Pb/^207^Pb	<2.37	>2.43	2.480 (1)	2.490 (1)

Note *: after Zhang^18^, **: (number of sample, one of the results of the granite samples in the table is after Wang^19^.

Lead isotope of the deposit’s middle-lower Triassic marbles in Table 12 is similar to that of the upper crust in Table 13. Lead isotope of the lower Permian metamorphosed basalt as well as that of pyrite in the basalt are between those of the mantle and the lower crust. Likewise, lead isotope of the granite of the Indo Chinese epoch is similar to that of the upper crust, though it was possibly contaminated with the lead from the crust.

In comparison to Table 14, it can be seen that lead isotopes of the granite at the Niubeishan outside the deposit and those of the wall rocks at the deposit, including slate, schist, marble and metamorphosed basalt as well as the pyrite in the basalt, are similar to that of the post Cambrian rocks.

**Figure 9:**
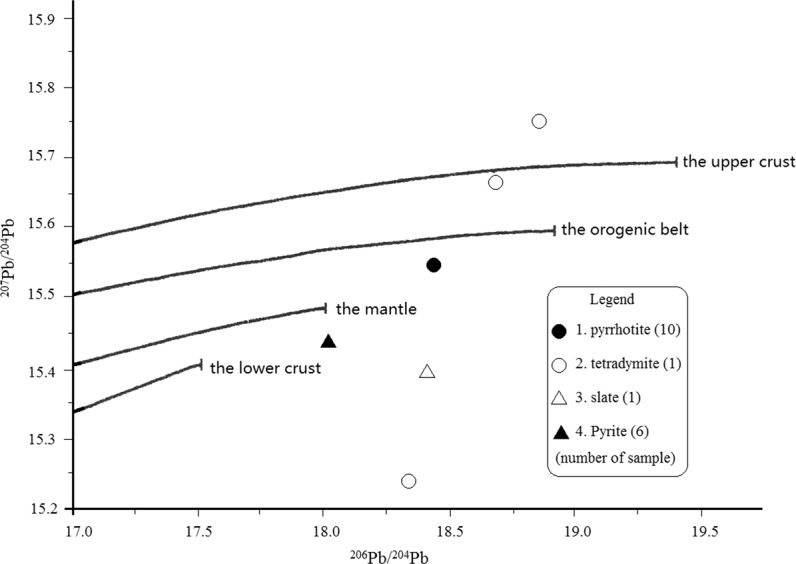
^207^Pb/^204^Pb - ^206^Pb/^204^Pb diagram of rocks/minerals from the study area.

As demonstrated in Tables 11, 12 and 13, the isotopes of pyrite and pyrrhotite of the same metallogenic epoch of the deposit are similar to each other and similar to that of the mantle, indicating that the ores’ leads are derived from the mantle. Lead isotopes of the tetradymite formed in the late metallogenic epoch are both similar to and different from that of pyrite and pyrrhotite formed in the early metallogenic epoch of the deposit, implying that lead isotopes of the late metallogenic epoch are mixtures of lead from both the mantle and the crust (Fig. 9).

Table 11 clearly shows that lead isotopes of tetradymite from different ore veins are different from each other. This likely implies that:

- Emplacement of the ore bodies completed in such a short period of time that lead isotopes in the ore did not have sufficient time to homogenize;
- When quickly uplifting and emplacing, the ore bodies rose in the form of ore pulp, during which lead isotopes from the crust were captured and mixed with the original mantle derived isotopes. This means that the lead isotope compositions can be used to determine the metallogenic mechanism of mineral deposits.

It is interesting to note that lead isotope of tetradymite from Ore vein #I-10 (series #22 sample in Table 11) is different from that of the series #24 sample collected at the footwall of Ore vein #I-10 in the same table. This may further confirm that tellurium ore bodies emplaced in the form of ore magma in a very quick process, during which captured and mixed leads from both the mantle and crust and made compositions of the lead isotopes very complicated.

It can be concluded on a preliminary basis that lead isotopes of the research area consist of more radioactive lead and are very inhomogeneous. The lead isotopes of the wall rocks slate, schist and metamorphosed basalt as well as the pyrite in the basalt are mantle derived, though overlapped with characteristics of lead of the later geological events. Lead isotopes of both pyrite and pyrrhotite are close to each other and mantle derived, while that of tetradymite is different from the former, indicating that they formed in different metallogenic epochs. Lead isotope compositions reveal that tellurium ore bodies emplaced in the form of ore magma in a quick process. Finally, lead of the deposit was mainly from the mantle but also partially captured some lead from the crust.

## Conclusions

- Chemical formula of the tetradymite from the Dashuigou deposit is Bi_2.00_Te_1.89_S_1.00_, which contains more Te but less S compared to the tetradymite with a chemical formula of Bi_2.00_Te_1.65_S_1.35_ on the JCPDS card in 1979, indicating that the Dashuigou tetradymite was formed in an Te-rich but S-poor environment.
- The negative values of the Dashuigou tetradymite’s pyroelectricity indicate that it formed in an environment with low sulfur fugacity.
- The sulfur isotope δ^34^S of both single sulfide minerals including tetradymite from different veins of different metallogenic epochs, and the general total sulfur isotope components of the deposit’s metallogenic hydrothermal solutions, vary within a very narrow scope and a very small range close to 0.0‰, indicating that the deposit’s sulfur is similar to that of meteorites and may be derived from the mantle.
- Lead isotopes of samples from the research area consist of more radioactive lead and are very inhomogeneous; lead isotopes of the wall rocks slate, schist and metamorphosed basalt as well as the pyrite in the basalt are mantle derived, though overlapped with characteristics of lead of the later geological events; lead isotopes of both pyrite and pyrrhotite are close to each other and mantle derived, while that of tetradymite is different from the former, demonstrating that they formed in different metallogenic epochs; lead isotope compositions reveal that tellurium ore bodies emplaced in a quick process mainly in the form of ore magma; lead of the deposit is mainly from the mantle but captured some other lead from the crust.

## Data Availability

The data that support the findings of this study is available from the authors upon reasonable request; see authors’ contributions for specific data sets.
